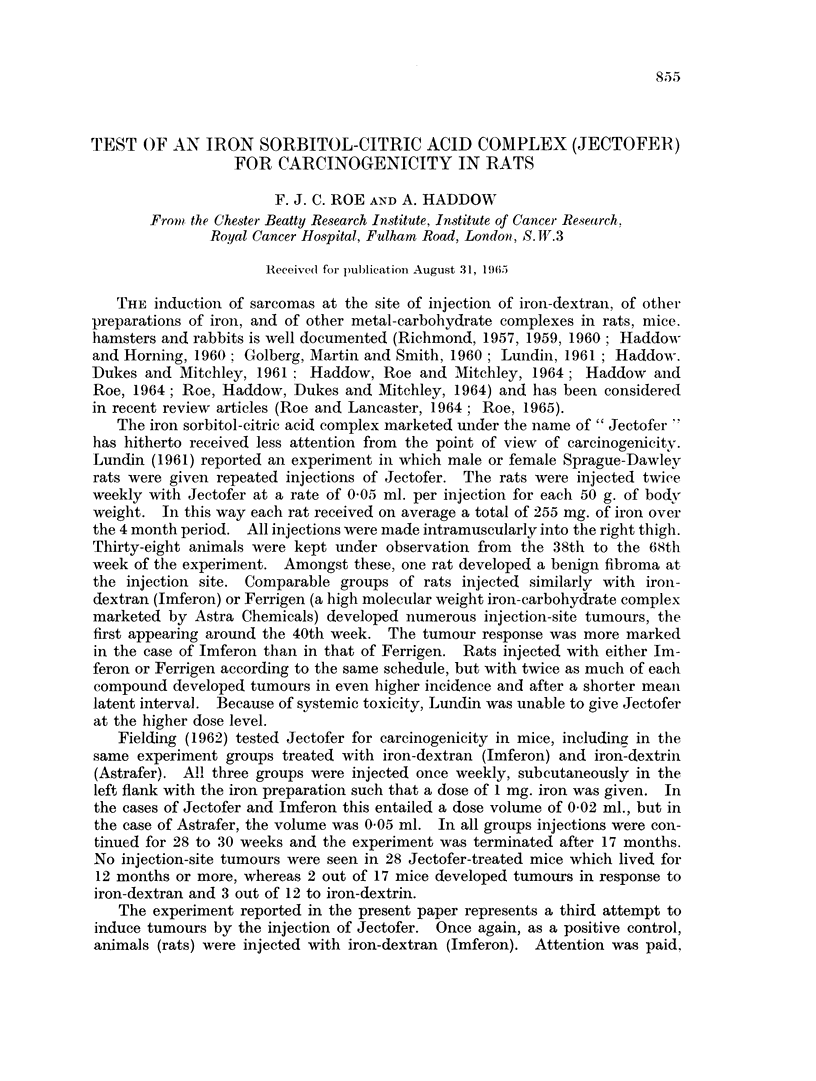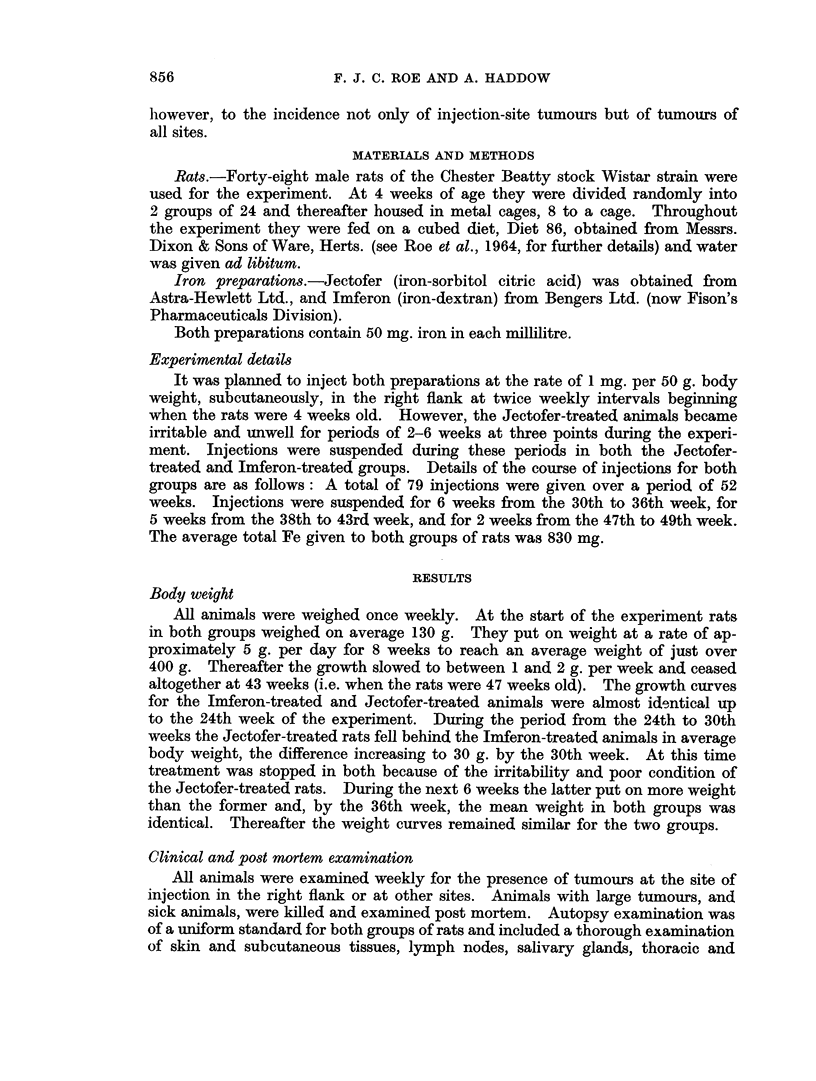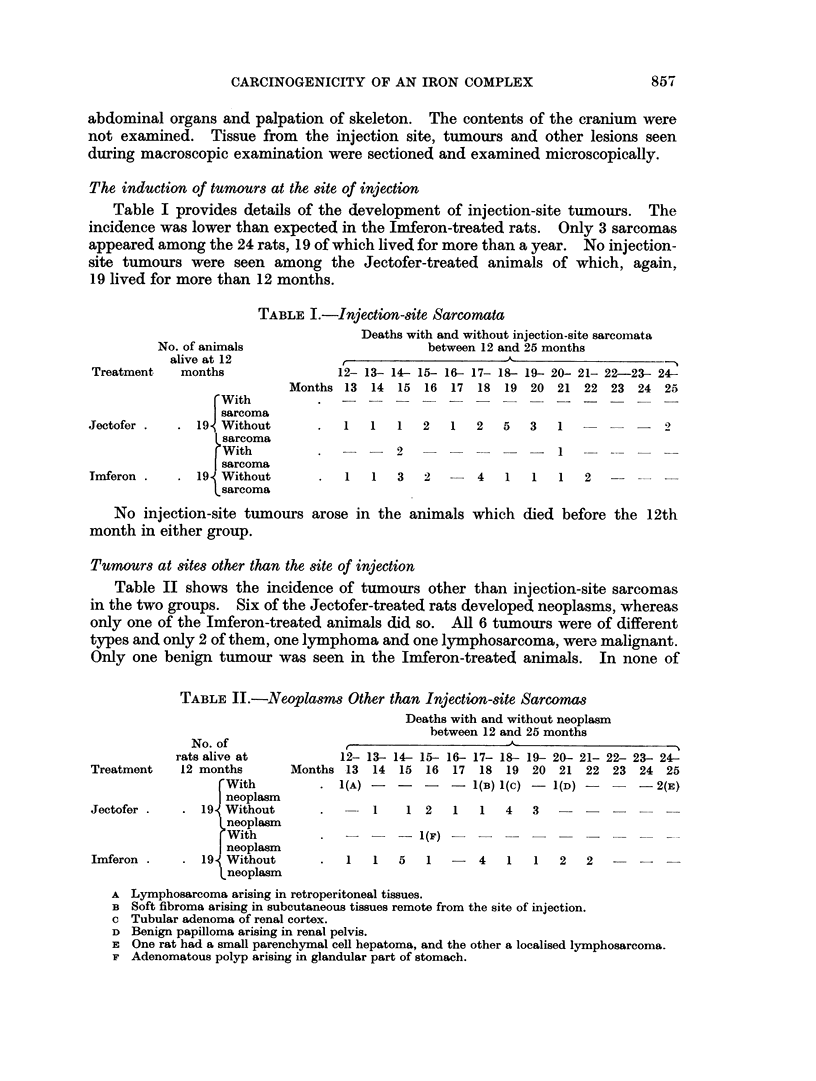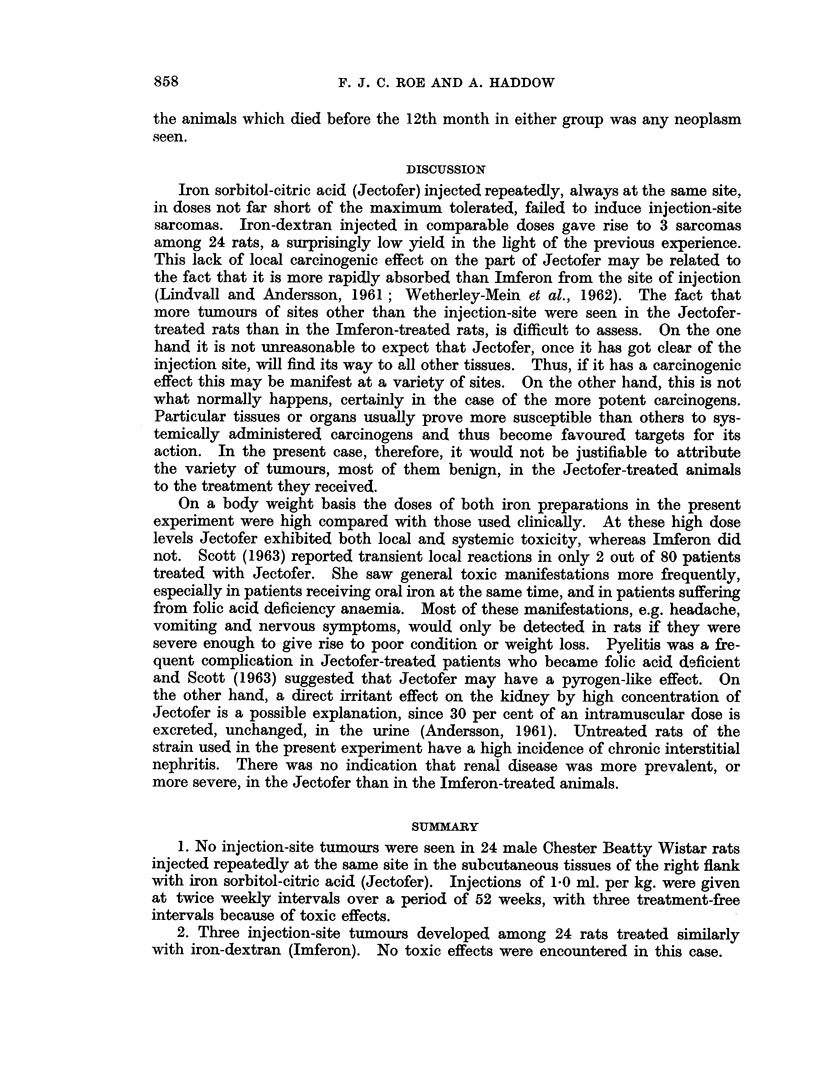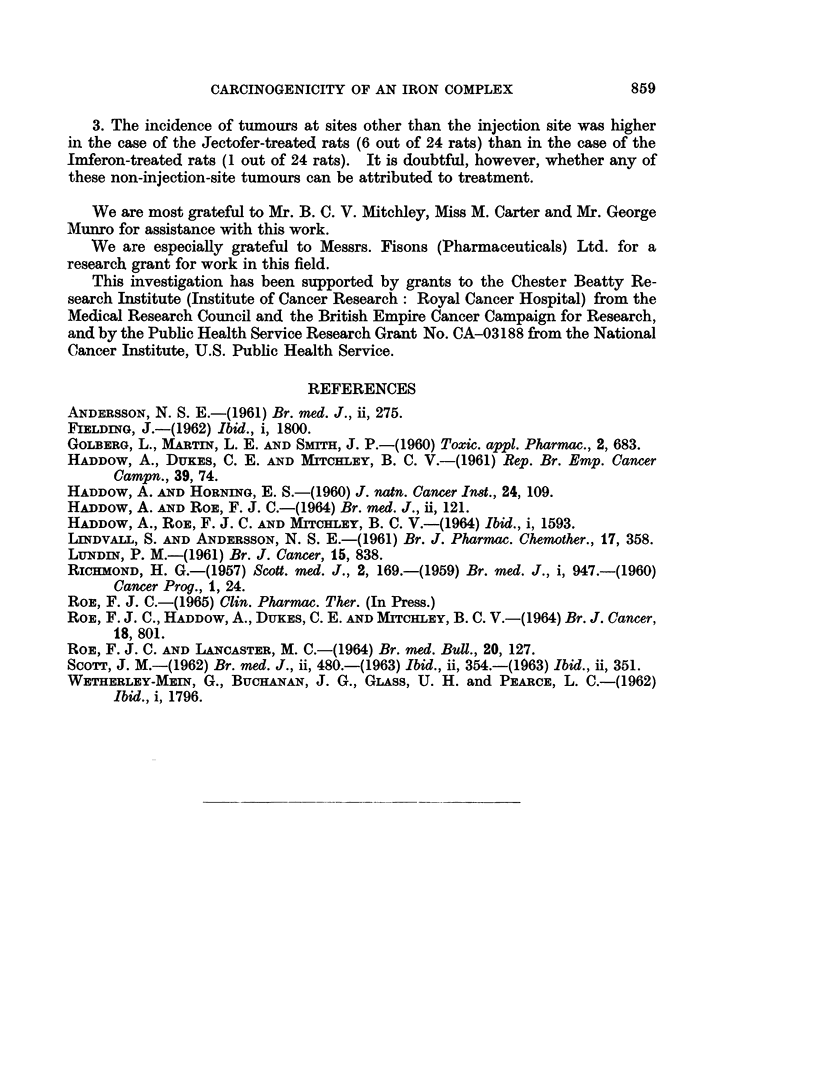# Test of an iron sorbitol-citric acid complex (Jectofer) for carcinogenicity in rats.

**DOI:** 10.1038/bjc.1965.98

**Published:** 1965-12

**Authors:** F. J. Roe, A. Haddow


### TEST OF AN IRON SORBITOL-CITRIC ACID COMPLEX (JECTOFER) FOR CARCINOGENICITY IN RATS

F. J. C. ROE AND A. HADDOW

*From the Chester Beatty Research Institute, Institute of Cancer Research, Royal Cancer Hospital, Fulham Road, London, S.W.3*

Received for publication August 31, 1965

THE induction of sarcomas at the site of injection of iron-dextran, of other preparations of iron, and of other metal-carbohydrate complexes in rats, mice, hamsters and rabbits is well documented (Richmond, 1957, 1959, 1960; Haddow and Horning, 1960; Golberg, Martin and Smith, 1960; Lundin, 1961; Haddow, Dukes and Mitchley, 1961; Haddow, Roe and Mitchley, 1964; Haddow and Roe, 1964; Roe, Haddow, Dukes and Mitchley, 1964) and has been considered in recent review articles (Roe and Lancaster, 1964; Roe, 1965).

The iron sorbitol-citric acid complex marketed under the name of "Jectofer" has hitherto received less attention from the point of view of carcinogenicity. Lundin (1961) reported an experiment in which male or female Sprague-Dawley rats were given repeated injections of Jectofer. The rats were injected twice weekly with Jectofer at a rate of 0·05 ml. per injection for each 50 g. of body weight. In this way each rat received on average a total of 255 mg. of iron over the 4 month period. All injections were made intramuscularly into the right thigh. Thirty-eight animals were kept under observation from the 38th to the 68th week of the experiment. Amongst these, one rat developed a benign fibroma at the injection site. Comparable groups of rats injected similarly with iron-dextran (Imferon) or Ferrigen (a high molecular weight iron-carbohydrate complex marketed by Astra Chemicals) developed numerous injection-site tumours, the first appearing around the 40th week. The tumour response was more marked in the case of Imferon than in that of Ferrigen. Rats injected with either Imferon or Ferrigen according to the same schedule, but with twice as much of each compound developed tumours in even higher incidence and after a shorter mean latent interval. Because of systemic toxicity, Lundin was unable to give Jectofer at the higher dose level.

Fielding (1962) tested Jectofer for carcinogenicity in mice, including in the same experiment groups treated with iron-dextran (Imferon) and iron-dextrin (Astrafer). All three groups were injected once weekly, subcutaneously in the left flank with the iron preparation such that a dose of 1 mg. iron was given. In the cases of Jectofer and Imferon this entailed a dose volume of 0·02 ml., but in the case of Astrafer, the volume was 0·05 ml. In all groups injections were continued for 28 to 30 weeks and the experiment was terminated after 17 months. No injection-site tumours were seen in 28 Jectofer-treated mice which lived for 12 months or more, whereas 2 out of 17 mice developed tumours in response to iron-dextran and 3 out of 12 to iron-dextrin.

The experiment reported in the present paper represents a third attempt to induce tumours by the injection of Jectofer. Once again, as a positive control, animals (rats) were injected with iron-dextran (Imferon). Attention was paid, however, to the incidence not only of injection-site tumours but of tumours of all sites.

### MATERIALS AND METHODS

*Rats.*—Forty-eight male rats of the Chester Beatty stock Wistar strain were used for the experiment. At 4 weeks of age they were divided randomly into 2 groups of 24 and thereafter housed in metal cages, 8 to a cage. Throughout the experiment they were fed on a cubed diet, Diet 86, obtained from Messrs. Dixon & Sons of Ware, Herts. (see Roe *et al.*, 1964, for further details) and water was given *ad libitum.*

*Iron preparations.*—Jectofer (iron-sorbitol citric acid) was obtained from Astra-Hewlett Ltd., and Imferon (iron-dextran) from Bengers Ltd. (now Fison's Pharmaceuticals Division).

Both preparations contain 50 mg. iron in each millilitre.

### *Experimental details*

It was planned to inject both preparations at the rate of 1 mg. per 50 g. body weight, subcutaneously, in the right flank at twice weekly intervals beginning when the rats were 4 weeks old. However, the Jectofer-treated animals became irritable and unwell for periods of 2–6 weeks at three points during the experiment. Injections were suspended during these periods in both the Jectofer-treated and Imferon-treated groups. Details of the course of injections for both groups are as follows : A total of 79 injections were given over a period of 52 weeks. Injections were suspended for 6 weeks from the 30th to 36th week, for 5 weeks from the 38th to 43rd week, and for 2 weeks from the 47th to 49th week. The average total Fe given to both groups of rats was 830 mg.

### RESULTS

### *Body weight*

All animals were weighed once weekly. At the start of the experiment rats in both groups weighed on average 130 g. They put on weight at a rate of approximately 5 g. per day for 8 weeks to reach an average weight of just over 400 g. Thereafter the growth slowed to between 1 and 2 g. per week and ceased altogether at 43 weeks (i.e. when the rats were 47 weeks old). The growth curves for the Imferon-treated and Jectofer-treated animals were almost identical up to the 24th week of the experiment. During the period from the 24th to 30th weeks the Jectofer-treated rats fell behind the Imferon-treated animals in average body weight, the difference increasing to 30 g. by the 30th week. At this time treatment was stopped in both because of the irritability and poor condition of the Jectofer-treated rats. During the next 6 weeks the latter put on more weight than the former and, by the 36th week, the mean weight in both groups was identical. Thereafter the weight curves remained similar for the two groups.

### *Clinical and post mortem examination*

All animals were examined weekly for the presence of tumours at the site of injection in the right flank or at other sites. Animals with large tumours, and sick animals, were killed and examined post mortem. Autopsy examination was of a uniform standard for both groups of rats and included a thorough examination of skin and subcutaneous tissues, lymph nodes, salivary glands, thoracic and abdominal organs and palpation of skeleton. The contents of the cranium were not examined. Tissue from the injection site, tumours and other lesions seen during macroscopic examination were sectioned and examined microscopically.

*The induction of tumours at the site of injection*

Table I provides details of the development of injection-site tumours. The incidence was lower than expected in the Imferon-treated rats. Only 3 sarcomas appeared among the 24 rats, 19 of which lived for more than a year. No injection-site tumours were seen among the Jectofer-treated animals of which, again, 19 lived for more than 12 months.

TABLE I.—*Injection-site Sarcomata*

| Treatment | No. of animals alive at 12 months | | Deaths with and without injection-site sarcomata between 12 and 25 months | | | | | | | | | | | | |
|---|---|---|---|---|---|---|---|---|---|---|---|---|---|---|---|
| | | Months | 12–13 | 13–14 | 14–15 | 15–16 | 16–17 | 17–18 | 18–19 | 19–20 | 20–21 | 21–22 | 22–23 | 23–24 | 24–25 |
| Jectofer | 19 | With sarcoma | — | — | — | — | — | — | — | — | — | — | — | — | — |
| | | Without sarcoma | 1 | 1 | 1 | 2 | 1 | 2 | 5 | 3 | 1 | — | — | — | 2 |
| Imferon | 19 | With sarcoma | — | — | 2 | — | — | — | — | — | 1 | — | — | — | — |
| | | Without sarcoma | 1 | 1 | 3 | 2 | — | 4 | 1 | 1 | 1 | 2 | — | — | — |

No injection-site tumours arose in the animals which died before the 12th month in either group.

*Tumours at sites other than the site of injection*

Table II shows the incidence of tumours other than injection-site sarcomas in the two groups. Six of the Jectofer-treated rats developed neoplasms, whereas only one of the Imferon-treated animals did so. All 6 tumours were of different types and only 2 of them, one lymphoma and one lymphosarcoma, were malignant. Only one benign tumour was seen in the Imferon-treated animals. In none of

TABLE II.—*Neoplasms Other than Injection-site Sarcomas*

| Treatment | No. of rats alive at 12 months | | Deaths with and without neoplasm between 12 and 25 months | | | | | | | | | | | | |
|---|---|---|---|---|---|---|---|---|---|---|---|---|---|---|---|
| | | Months | 12–13 | 13–14 | 14–15 | 15–16 | 16–17 | 17–18 | 18–19 | 19–20 | 20–21 | 21–22 | 22–23 | 23–24 | 24–25 |
| Jectofer | 19 | With neoplasm | 1(A) | — | — | — | — | 1(B) | 1(C) | — | 1(D) | — | — | — | 2(E) |
| | | Without neoplasm | — | 1 | 1 | 2 | 1 | 1 | 4 | 3 | — | — | — | — | — |
| Imferon | 19 | With neoplasm | — | — | — | 1(F) | — | — | — | — | — | — | — | — | — |
| | | Without neoplasm | 1 | 1 | 5 | 1 | — | 4 | 1 | 1 | 2 | 2 | — | — | — |

A Lymphosarcoma arising in retroperitoneal tissues.
B Soft fibroma arising in subcutaneous tissues remote from the site of injection.
C Tubular adenoma of renal cortex.
D Benign papilloma arising in renal pelvis.
E One rat had a small parenchymal cell hepatoma, and the other a localised lymphosarcoma.
F Adenomatous polyp arising in glandular part of stomach.

the animals which died before the 12th month in either group was any neoplasm seen.

### DISCUSSION

Iron sorbitol-citric acid (Jectofer) injected repeatedly, always at the same site, in doses not far short of the maximum tolerated, failed to induce injection-site sarcomas. Iron-dextran injected in comparable doses gave rise to 3 sarcomas among 24 rats, a surprisingly low yield in the light of the previous experience. This lack of local carcinogenic effect on the part of Jectofer may be related to the fact that it is more rapidly absorbed than Imferon from the site of injection (Lindvall and Andersson, 1961; Wetherley-Mein *et al.*, 1962). The fact that more tumours of sites other than the injection-site were seen in the Jectofer-treated rats than in the Imferon-treated rats, is difficult to assess. On the one hand it is not unreasonable to expect that Jectofer, once it has got clear of the injection site, will find its way to all other tissues. Thus, if it has a carcinogenic effect this may be manifest at a variety of sites. On the other hand, this is not what normally happens, certainly in the case of the more potent carcinogens. Particular tissues or organs usually prove more susceptible than others to systemically administered carcinogens and thus become favoured targets for its action. In the present case, therefore, it would not be justifiable to attribute the variety of tumours, most of them benign, in the Jectofer-treated animals to the treatment they received.

On a body weight basis the doses of both iron preparations in the present experiment were high compared with those used clinically. At these high dose levels Jectofer exhibited both local and systemic toxicity, whereas Imferon did not. Scott (1963) reported transient local reactions in only 2 out of 80 patients treated with Jectofer. She saw general toxic manifestations more frequently, especially in patients receiving oral iron at the same time, and in patients suffering from folic acid deficiency anaemia. Most of these manifestations, e.g. headache, vomiting and nervous symptoms, would only be detected in rats if they were severe enough to give rise to poor condition or weight loss. Pyelitis was a frequent complication in Jectofer-treated patients who became folic acid deficient and Scott (1963) suggested that Jectofer may have a pyrogen-like effect. On the other hand, a direct irritant effect on the kidney by high concentration of Jectofer is a possible explanation, since 30 per cent of an intramuscular dose is excreted, unchanged, in the urine (Andersson, 1961). Untreated rats of the strain used in the present experiment have a high incidence of chronic interstitial nephritis. There was no indication that renal disease was more prevalent, or more severe, in the Jectofer than in the Imferon-treated animals.

### SUMMARY

1. No injection-site tumours were seen in 24 male Chester Beatty Wistar rats injected repeatedly at the same site in the subcutaneous tissues of the right flank with iron sorbitol-citric acid (Jectofer). Injections of 1·0 ml. per kg. were given at twice weekly intervals over a period of 52 weeks, with three treatment-free intervals because of toxic effects.

2. Three injection-site tumours developed among 24 rats treated similarly with iron-dextran (Imferon). No toxic effects were encountered in this case.

3. The incidence of tumours at sites other than the injection site was higher in the case of the Jectofer-treated rats (6 out of 24 rats) than in the case of the Imferon-treated rats (1 out of 24 rats). It is doubtful, however, whether any of these non-injection-site tumours can be attributed to treatment.

We are most grateful to Mr. B. C. V. Mitchley, Miss M. Carter and Mr. George Munro for assistance with this work.

We are especially grateful to Messrs. Fisons (Pharmaceuticals) Ltd. for a research grant for work in this field.

This investigation has been supported by grants to the Chester Beatty Research Institute (Institute of Cancer Research : Royal Cancer Hospital) from the Medical Research Council and the British Empire Cancer Campaign for Research, and by the Public Health Service Research Grant No. CA-03188 from the National Cancer Institute, U.S. Public Health Service.

### REFERENCES

ANDERSSON, N. S. E.—(1961) *Br. med. J.*, ii, 275.

FIELDING, J.—(1962) *Ibid.*, i, 1800.

GOLBERG, L., MARTIN, L. E. AND SMITH, J. P.—(1960) *Toxic. appl. Pharmac.*, **2**, 683.

HADDOW, A., DUKES, C. E. AND MITCHLEY, B. C. V.—(1961) *Rep. Br. Emp. Cancer Campn.*, **39**, 74.

HADDOW, A. AND HORNING, E. S.—(1960) *J. natn. Cancer Inst.*, **24**, 109.

HADDOW, A. AND ROE, F. J. C.—(1964) *Br. med. J.*, ii, 121.

HADDOW, A., ROE, F. J. C. AND MITCHLEY, B. C. V.—(1964) *Ibid.*, i, 1593.

LINDVALL, S. AND ANDERSSON, N. S. E.—(1961) *Br. J. Pharmac. Chemother.*, **17**, 358.

LUNDIN, P. M.—(1961) *Br. J. Cancer*, **15**, 838.

RICHMOND, H. G.—(1957) *Scott. med. J.*, **2**, 169.—(1959) *Br. med. J.*, i, 947.—(1960) *Cancer Prog.*, **1**, 24.

ROE, F. J. C.—(1965) *Clin. Pharmac. Ther.* (In Press.)

ROE, F. J. C., HADDOW, A., DUKES, C. E. AND MITCHLEY, B. C. V.—(1964) *Br. J. Cancer*, **18**, 801.

ROE, F. J. C. AND LANCASTER, M. C.—(1964) *Br. med. Bull.*, **20**, 127.

SCOTT, J. M.—(1962) *Br. med. J.*, ii, 480.—(1963) *Ibid.*, ii, 354.—(1963) *Ibid.*, ii, 351.

WETHERLEY-MEIN, G., BUCHANAN, J. G., GLASS, U. H. and PEARCE, L. C.—(1962) *Ibid.*, i, 1796.